# Long-term outcomes of patients evacuated from hospitals near the Fukushima Daiichi nuclear power plant after the Great East Japan Earthquake

**DOI:** 10.1371/journal.pone.0195684

**Published:** 2018-04-17

**Authors:** Yutaka Igarashi, Takashi Tagami, Jun Hagiwara, Takahiro Kanaya, Norihiro Kido, Mariko Omura, Ryoichi Tosa, Hiroyuki Yokota

**Affiliations:** 1 Department of Emergency and Critical Care Medicine, Nippon Medical School Hospital, Tokyo, Japan; 2 Health Services and Systems Research, Duke-NUS Medical School, Singapore; 3 Department of Emergency and Critical Care Medicine, Nippon Medical School Tama Nagayama Hospital, Tokyo, Japan; 4 Department of Emergency and Critical Care Medicine, Nippon Medical School Musashi Kosugi Hospital, Kanagawa, Japan; 5 Department of Emergency and Critical Care Medicine, Aizu Chuo Hospital, Fukushima, Japan; University of South Carolina, UNITED STATES

## Abstract

**Introduction:**

After the accident of the Fukushima Daiichi nuclear power plant due to the Great East Japan Earthquake in March 2011, the Japanese government issued a mandatory evacuation order for people living within a 20 km radius of the nuclear power plant. The aim of the current study was to investigate long-term outcomes of these patients and identify factors related to mortality.

**Materials and methods:**

Patients who were evacuated from hospitals near the Fukushima Daiichi nuclear power plant to the Aizu Chuo Hospital from 15 to 26 March, 2011 were included in this study. The following data were collected from medical records: age, sex, activities of daily life, hospital they were admitted in at the time of earthquake, distance between the facility and the nuclear power plant, reasons of evacuation and number of transfers. The patient outcomes were collected from medical records and/or investigated on the telephone in January 2012.

**Results:**

A total of 97 patients (28 men and 69 women) were transferred from 10 hospitals via ambulances or buses. No patients died or experienced exacerbation during transfer. Median age of the patients was 86 years. Of the total, 36 patients were not able to obey commands, 44 were bed-ridden and 61 were unable to sustain themselves via oral intake of food. Among 86 patients who were followed-up, 41 (48%) died at the end of 2011. Multiple-regression analysis showed that non-oral intake [Hazard Ratio (HR): 6.07, 95% Confidence interval (CI): 1.94–19.0] and male sex [HR: 8.35, 95% CI: 2.14–32.5] had significant impact on mortality.

**Conclusion:**

This study found that 48% of the evacuated patients died 9 months after the earthquake and they had significantly higher mortality rate than the nursing home residents. Non-oral intake and male sex had significant impact on mortality. These patients should be considered as especially vulnerable in case of hospital evacuation.

## Introduction

The Great East Japan Earthquake occurred on 11 March 2011 and devastated a large area of eastern Japan. The Fukushima Daiichi nuclear power plant caused nuclear meltdowns, hydrogen-air explosions and the release of radioactive material on 12 March. The Japanese government issued a mandatory evacuation order for those living within a 20 km radius of the nuclear power plant and indoor shelter and voluntary evacuation instructions for residents within a 20–30 km zone. The hospitalised patients and nursing home residents within this area were also evacuated. In one such instance, approximately 50 patients transferred from a hospital to a high school gymnastic hall died during transfer or soon after arrival[1].

Several reports suggested that nursing home residents and hospitalised patients evacuated from facilities during disasters experience higher mortality [2, 3]. It has also been reported that nursing home residents experienced aggravation of chronic diseases due to the Great East Japan Earthquake[4]. However, little is known about the long-term outcomes and the factors related to mortality with respect to evacuated, hospitalised patients of this disaster [1, 5]. The aim of the current study was to investigate long-term outcomes of these patients and identify factors related to poor outcomes.

## Materials and methods

### Ethical statement

All photographs were processed to protect the anonymity of all the participants of this study, and all individually identifiable data were removed to respect the patients’ privacy. The individual in this manuscript has given written informed consent (as outlined in PLOS consent form) to publish these case details. The Ethics Committee of the Aizu Chuo Hospital waived the requirement for informed consent and approved this study.

### Settings

**Fig 1:**
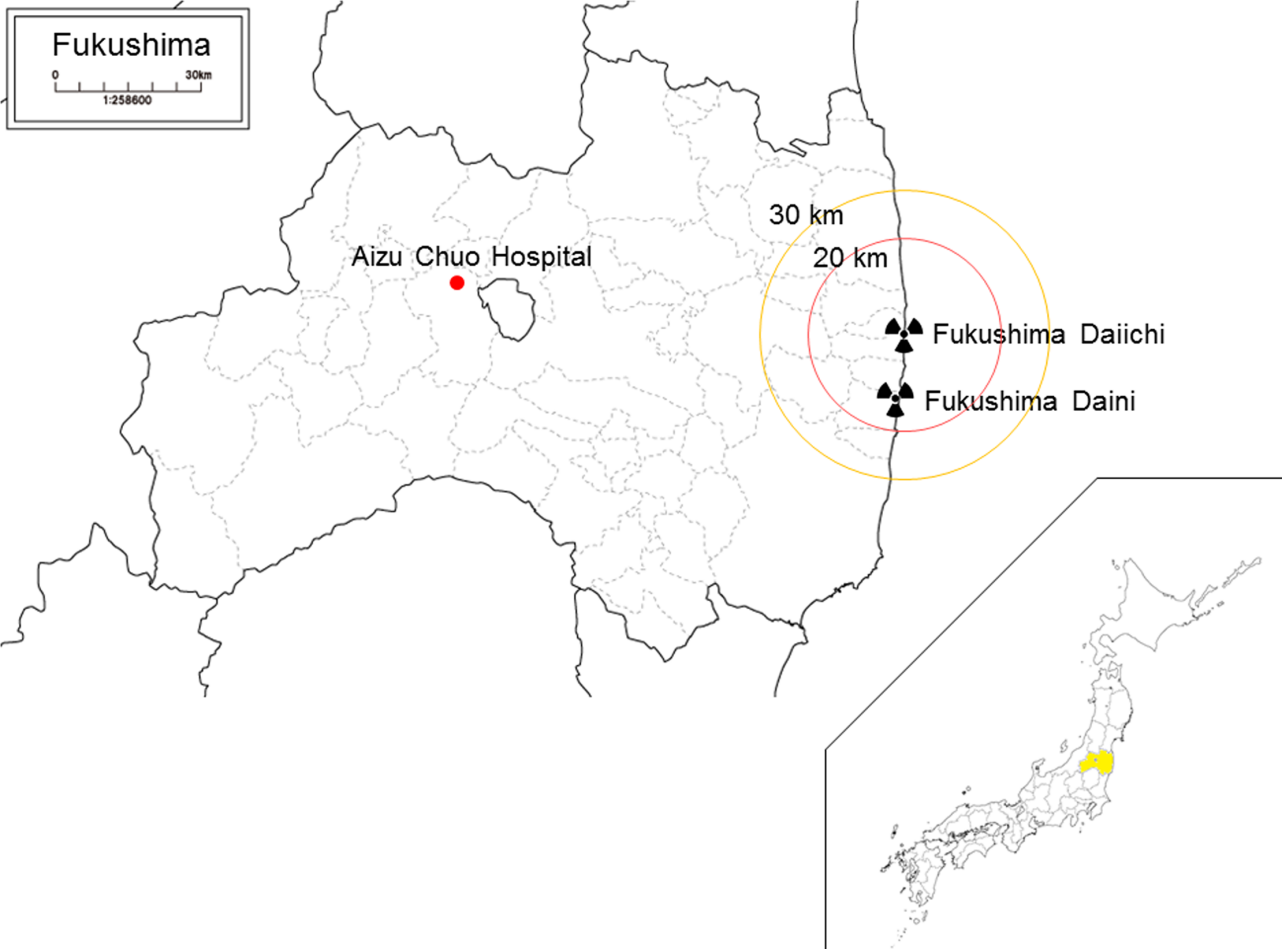
Location of the Fukushima Daiichi nuclear power plant and the Aizu Chuo Hospital.

The Aizu Chuo Hospital is located approximately 100 km west of the Fukushima Daiichi nuclear power plant, Fukushima, Japan. It is a tertiary-care hospital with 898 beds and the largest emergency room in the Aizu region, which comprises suburban/rural communities and covers 5420 km^2^ with approximately 300,000 residents[6] (Fig 1). It is also one of the seven disaster base hospitals in Fukushima, which play a vital role in intensive treatment of severely injured patients, regional transportation of patients, and deployment of disaster medical assistance teams during a disaster. The Aizu Chuo Hospital experienced relatively lesser structural damage and limited radiation exposure compared with other areas in the prefecture. However, there were shortages in food and medical supplies; as on 13 March 2011. Food for patients was expected to last only for three days, oxygen for four days, drug injections for seven days, blood tests for seven days and heavy oil for in-house power generation for 12 days. Because tsunami-affected areas were given priority over the Aizu region in the context of medical supplies and food, the hospital adopted a policy that patients with planned surgeries and complete medical cheque-ups were to be discharged as soon as possible and their procedures postponed. Seven patients requiring post-operative care were transferred from the other hospital in Aizu region and three patients requiring acute medical care were transferred from tsunami-affected areas via helicopter[7]. Moreover, patients evacuated from hospitals near the nuclear power plant were to be accepted if requested by headquarters for disaster control in the Fukushima prefecture.

### Plan and practice of receiving evacuated patients

A decision was made that 48 patients would be transferred from temporary evacuated patient locations on 18^th^ March. We made preparations to accept the patients as efficiently as possible to prevent death and exacerbation during transfer; first, radiation exposure of patients was measured; second, if their radiation exposure was within normal limits, they were transferred to triage tents; if their radiation exposure was above the limit, they were decontaminated; third, vital signs were measured by nurses, medical records were checked and medications were ordered by doctors at the triage point; fourth, chest X-ray, electrocardiography and blood tests were performed; finally, the patients were transferred to the ward by nurses. We ensured that different patient traffic routes did not overlap with each other.

**Fig 2:**
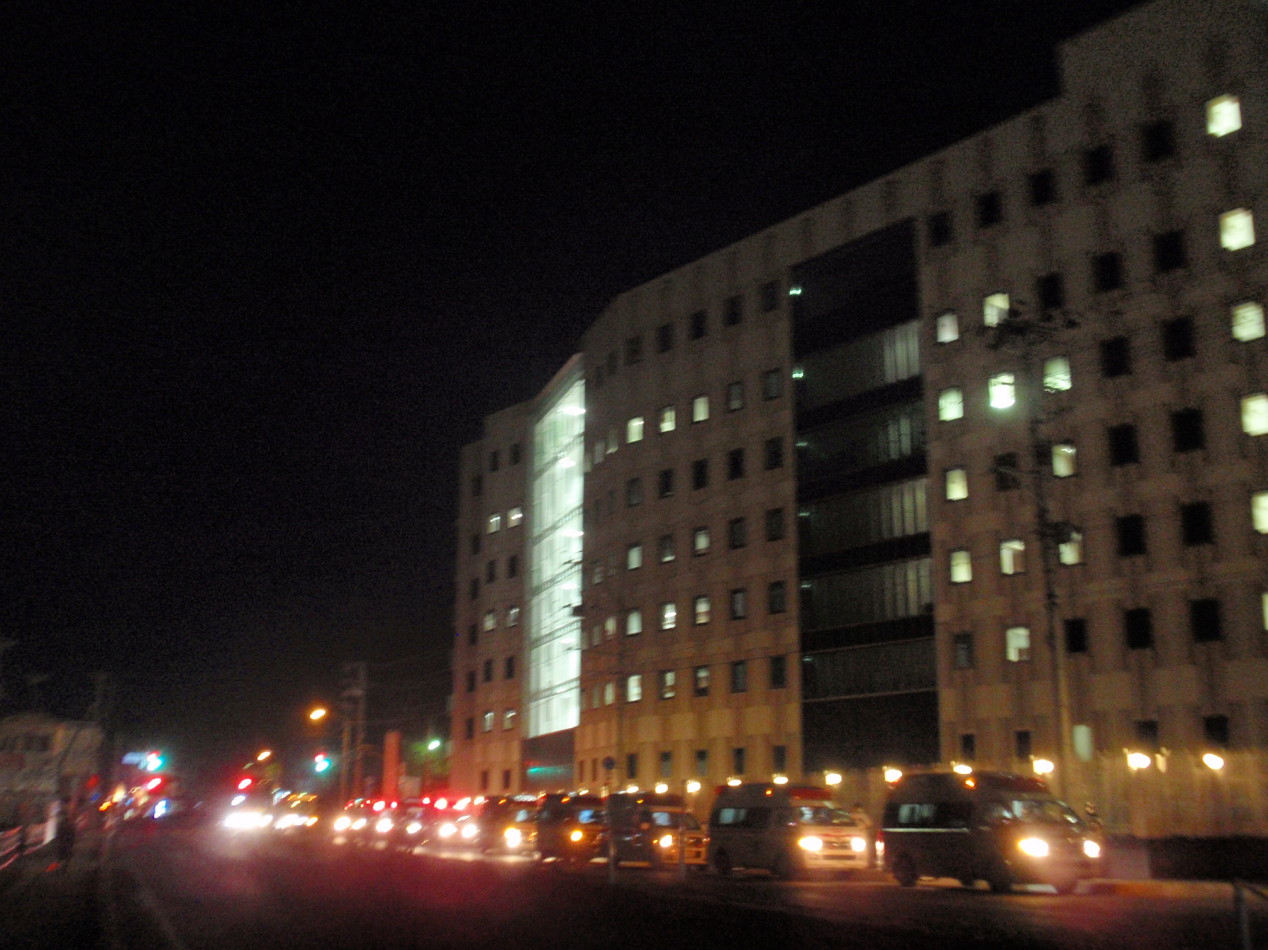
Ambulances forming a line along the street in front of the hospital.

**Fig 3:**
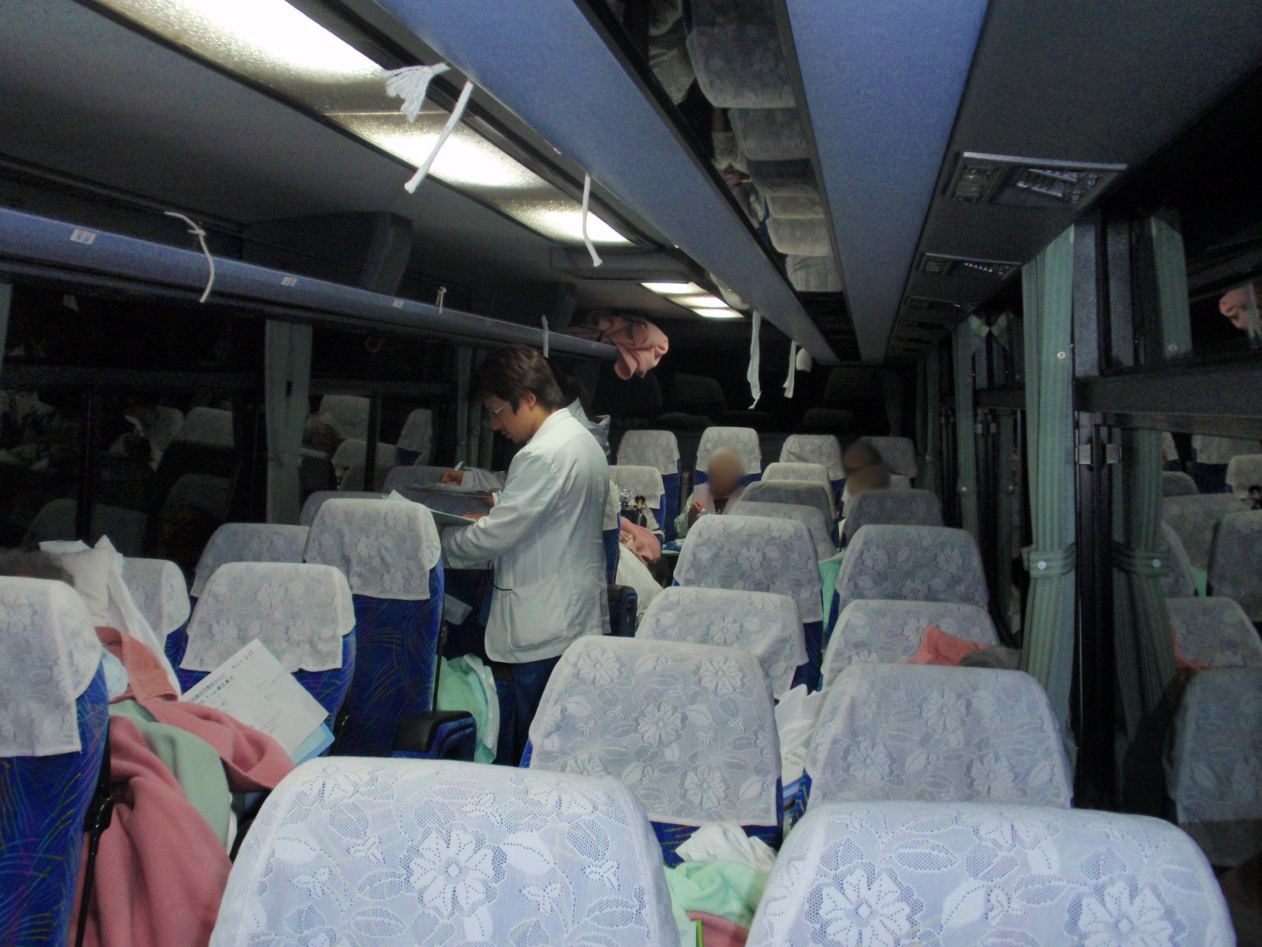
An emergency doctor performing triage in the bus after patient arrival.

**Fig 4:**
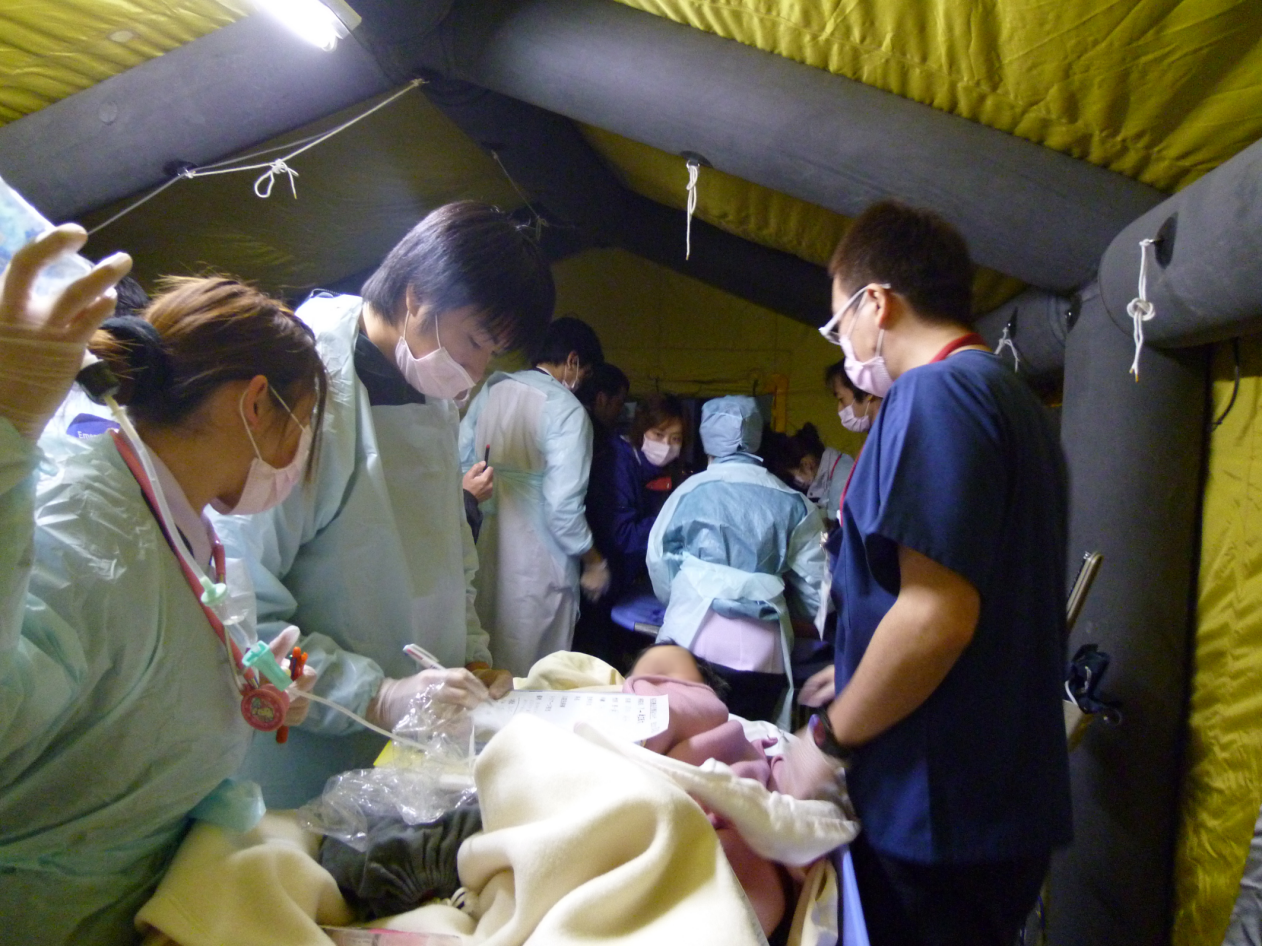
An emergency doctor and a nurse examining a patient in a triage tent.

Patient lists were sent to us via facsimile before transfer. All patients were transferred via ambulances or tourist buses. Several ambulances were assembled as emergency fire response teams and patients were transferred by emergency medical technicians. Ambulances were lined along the street in front of the hospital (Fig 2). Other patients were transferred via bus with a resident physician and a medical student, although several of them were unable to sit up and had to lie down on the seats. An emergency physician performed medical triage in the bus, but no patient was assessed to be in an emergency category (Fig 3). Because most patients were unable to walk as they were unconsciousness, based on expertise, the doctors specializing in emergency medicine checked the patients’ respiration and circulation immediately upon arrival; no particular triage tool or protocol was used. No patient was exposed to radiation above the limit of radiation exposure. Additionally, no patient was suspected of internal radiation exposure or decontaminated before arriving at the hospital. The patient’s vital signs were measured by nurses, once they were carried out from the ambulances, buses, or taxis by the medical staff. Personalized treatments and medications were further provided by the doctors in charge at the triage point, and the patients were then transferred to appropriate medical wards. (Fig 4). A few patients did not have their medical records and had very little information; one patient did not have any information and, therefore, only her name was written on her forearm with an oil-based marker. No patients died or experienced exacerbation during the transfer.

### Study patients and data collection

Patients evacuated from hospitals near the Fukushima Daiichi nuclear power plant and transferred to the Aizu Chuo Hospital 15 to 26 March, 2011 were included in this study. Patient data were collected from their medical records retrospectively, and all data were completely anonymised. The following data were collected from all patients: age, sex, daily activities, the hospital where the patients were admitted at the time of earthquake, the distance between the facility and nuclear power plant, the reasons of evacuation, and the number of transfers. The patient outcomes were collected from medical records and/or investigated on telephone in January 2012.

### Comparison of mortality of nursing home residents

Previous research reported that 328 residents from 5 nursing homes in Minamisoma city, which were located within 20–30 km away from the power plant, were evacuated, and 75 people died by the end of 2011 [3]. These residents represented 62% of all nursing home residents at the time of the earthquake. These data were used as control population because patients with chronic diseases were enrolled in this study and must have represented similar patients’ characteristics.

### Statistical analysis

Data are presented as mean values ± standardised deviation or as median, depending on the distribution normality of the variable. Chi-squared test or *t*-test was used to compare survival rate and mortality. Multiple regression analyses were performed. Age, sex, the hospital where the patients were admitted at the time of earthquake, the number of transfers, and non-oral intake were chosen as variables because these data were reported to influence mortality in previous studies. Statistical analyses were performed using StatFlex version 6.0 (Artech Co., Ltd., Osaka, Japan). A *p*-value of <0.05 was considered to be statistically significant.

## Results

**Fig 5:**
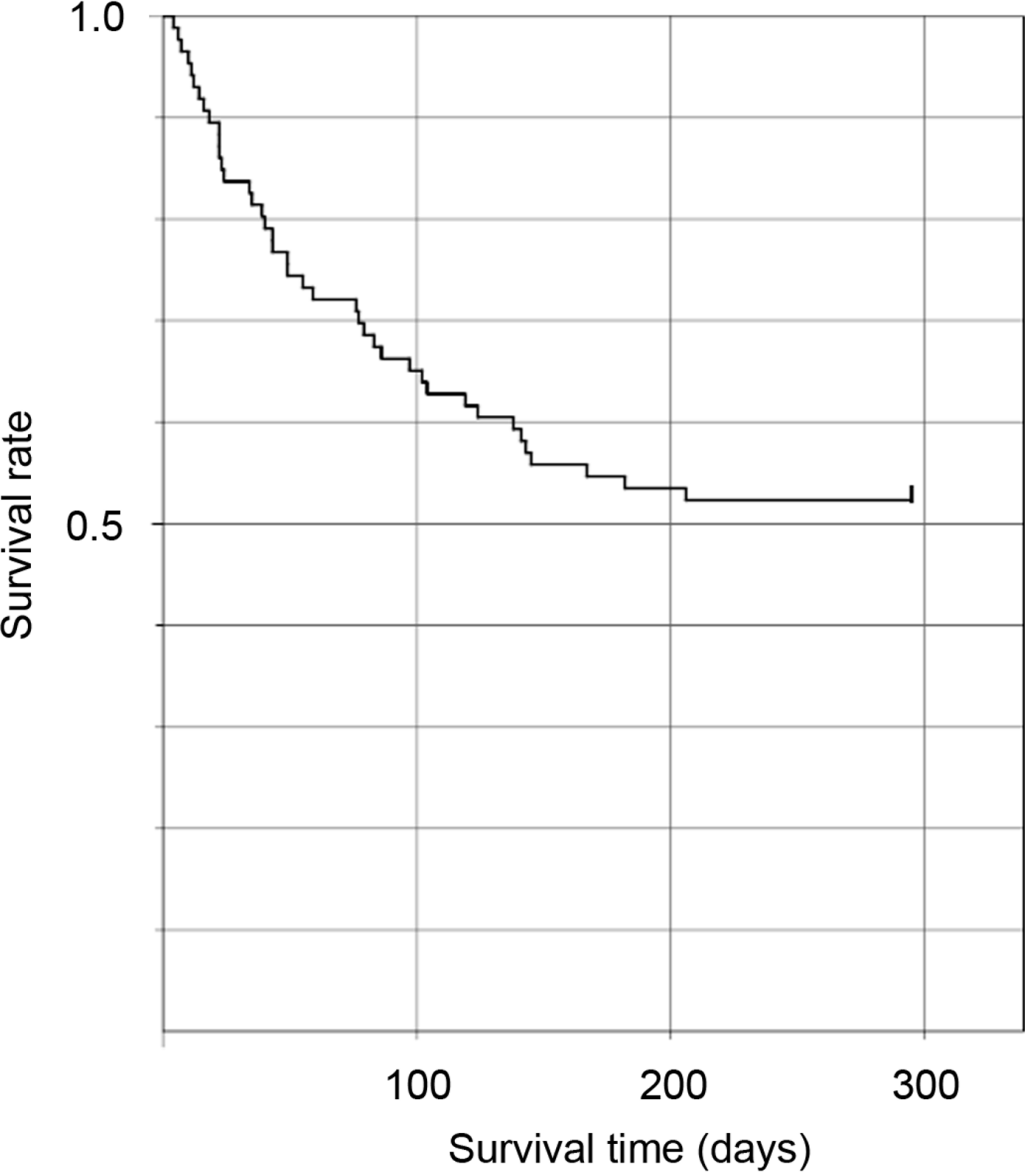
Post-earthquake survival for the patients evacuated from the hospitals near the Fukushima Daiichi nuclear power plant.

In total, 97 patients (28 men and 69 women) were transferred from 10 hospitals. Patient characteristics and outcomes are represented in Table 1. Median age of the patients was 86 years. Among these, 36 (37%) were not able to obey commands, 44 (45%) were bed-ridden and 61 (63%) were unable to sustain themselves via oral intake of food. At the time of the earthquake, 39 patients (40%) were admitted to facilities within 20 km of the nuclear plant, 49 patients (51%) to those within 20–30 km, and 9 patients (9%) to those beyond 30 km. Eighty-eight patients (91%) were evacuated. Fifty-six patients (58%) were transferred to our hospital via one hospital as a shelter, 30 patients (31%) via two temporary locations and 10 patients (10%) via three temporary locations. Fourteen patients (14%) died within a month of the disaster. Sixty-three patients (65%), who were in a stable condition, were transferred to other hospitals. Among the 97 patients who were evacuated, 86 could be followed-up. Among patients who were able to be followed-up, 41 (47%) died at the end of 2011 (Fig 5). Although 20 patients died among 52 patients with consciousness, 21 patients died among 34 patients with unconsciousness. The patients with unconsciousness had significantly higher mortality than those with consciousness (61.8% vs. 38.5%; *p* = 0.0344). Among 328 nursing home residents, 75 people died until the end of 2011. The mortality rate of the evacuated patients and nursing home residents were 47.7% and 22.8%, respectively. The evacuated patients had significantly higher mortality rate than the nursing home residents (*p* < 0.00001).

**Table 1:** Patient characteristics between survival and mortality (N = 86).

¤	Survival¤	Mortality¤	*P*-value¤
¤	(N = 45)¤	(N = 41)¤	¤
**Age (years)¤**	85 (79–90)¤	86 (81–90) ¤	0.50¤
**Sex¤**	¤	¤	0.0075¤
→ Male¤	7¤	17¤	¤
→ Female¤	38¤	24¤	¤
**Activity of daily life¤**	¤	¤	¤
→ Not able to obey command	13	21	0.03
→ Bed-ridden	18	22	0.20¤
→ Non-oral intake¤	22¤	33¤	0.0023¤
**Distance between the facility to be admitted and the nuclear power plant¤**	¤	¤	¤
→ <20 km¤	18¤	16¤	0.93¤
→ 20–30 km¤	23¤	22¤	0.81¤
→ >30 km¤	4¤	3¤	0.79¤
**Reason of evacuation¤**	¤	¤	0.79¤
→ Evacuation order¤	41¤	38¤	¤
→ Others¤	4¤	3¤	¤
**Number of transfer¤**	¤	¤	¤
→ One¤	26¤	24¤	0.94¤
→ Two¤	13¤	14¤	0.60¤
→ Three¤	6¤	3¤	0.62¤

Non-oral intake and male sex had significant impact on mortality but the other variables did not. Results of Multiple regression analysis are shown in Table 2.

**Table 2:** Multiple regression model of survival.

Variable¤	Hazard ratio¤	95% confidence interval¤	*P*-value¤
**Age¤**	1.05¤	0.98–1.12¤	0.16¤
**Sex¤**	8.35¤	2.14–32.5¤	0.0022¤
**Facility¤**	0.89¤	0.67–1.19¤	0.43¤
**Number of transfer¤**	1.05¤	0.52–2.09¤	0.90¤
**Non-oral intake¤**	6.07¤	1.94–19.0¤	0.0022¤

## Discussion

Forty-eight percent of patients evacuated after the Great East Japan Earthquake died within 9 months of the earthquake and had significantly higher mortality rates than that of the nursing home residents. Non-oral intake and male sex were significantly associated with mortality.

Various cases of evacuation have been reported during natural and man-made disasters, including hurricanes [8–18], cyclone [19, 20], storm [21, 22], wildfires [23, 24], earthquake [25–27], flooding [28–30], nuclear meltdown [5, 31], internal fire [32], bomb threats [33, 34], missiles [35] and chemical exposure [33]. During the disaster, it was known that hospital evacuation increases mortality and morbidity [2]. On comparing the differential mortality and morbidity among long-stay residents exposed to four hurricanes, including Katrina, with those of the same residents over the same period during the prior two non-hurricane years as controls, a cumulative total of 579 extra deaths and 544 extra hospitalisations were observed at 90 days after the hurricanes than those before the hurricanes. Evacuation increased the probability of death at 90 days after the hurricanes from 2.7% to 5.3%, and that of hospitalisation from 1.8% to 8.3%, independent of other factors[2]. One study concluded that the overall relative mortality risk before and after the earthquake was 2.683 among the nursing home residents evacuated after the Fukushima nuclear accident[3]. Moreover, the present study revealed that the mortality rate of evacuated patients was 48% at 9 months after the earthquake and was significantly higher than that of the nursing home residents.

Some facilities have reported that age and high-level care are factors that increase the risk of mortality[3]. Our study found that non-oral intake and male sex were factors increasing relative risk. Patients who need high-level care are likely to experience hypothermia, dehydration, or deterioration of underlying medical problems during and after transfer[1]. Patients who survived transfer experienced higher mortality over a long period, probably due to generalised stress and low quality of care at the evacuation site. Although generalised stress could have resulted in myocardial infarction over a long period [36–38], psychological stress could not have impacted mortality because more than half of the patients were unconscious during the transfer. Low quality of care may have been caused due to acceptance of several patients simultaneously and limited dissemination of medical information, although the hospital did not experience medical supply shortage or electricity and water outage.

Because unplanned and unprepared evacuation may result in higher mortality, hospital evacuation should be considered carefully. In the Great East Japan Earthquake, total loss of life expectancy of residents due to evacuation-related risks in rapid evacuation was much higher than that due to radiation in other scenarios[39]. Evacuation orders should, therefore, be well balanced with trade-offs against radiation risks. On the contrary, care should also be made available to the patients who have not been evacuated from a potentially contaminated zone. Other hospitals near the evacuated hospitals should be prepared not only for acutely ill patients but also for a possibly large number of patients evacuated.

This study was limited by three specific factors. First, according the Fukushima prefectural government, 1,333 patients were evacuated from 13 hospitals within a 30 km radius of the nuclear power plant. It was considered that several patients with acute illness were included in these groups; however, only patients with chronic diseases were enrolled in this study. The study results may not reflect the entire hospitalized patient population, and the mortality of this study may be under- or overestimated. Second, this study might also be biased in terms of patient selection. Only patients in a relatively good condition were transferred to this hospital. However, most patients in this study were evacuated via multiple hospitals, and this was not the initial evacuation for these patients because mortality of initial evacuation was 1.94 times higher than that of the subsequent evacuation[3]. Although it was difficult to assess the effect, we accepted and treated all patients as we were the only disaster based hospital in the Aizu region. Because many patients did not have their medical records and referral forms because of the haste and complexity of the evacuation, obtaining detailed history was difficult. Third, other medical data could not be added as a variable for the multiple regression analysis. Unmeasured confounding factors might have existed and led to a bias. This study also involved patients who survived the transfer and did not show risk factors for exacerbation.

## Conclusions

This study found that 48% of the evacuated patients died within 9 months of the Great East Japan earthquake and had significantly higher mortality rate than the nursing home residents. Non-oral intake and male sex had significant impact on mortality. These patients should be considered as especially vulnerable in case of hospital evacuation.

## Supporting information

S1 Table Individual patient data. (XLSX)
